# Sport-Related Effect on Knee Strength Profile during Puberty: Basketball vs. Soccer

**DOI:** 10.3390/jfmk8020057

**Published:** 2023-05-08

**Authors:** Vassilis Gerodimos, Konstantina Karatrantou, Christos Batatolis, Panagiotis Ioakimidis

**Affiliations:** Department of Physical Education and Sports Science, University of Thessaly, 42100 Trikala, Greece

**Keywords:** testing and evaluation, team sports, developmental ages, isokinetic, injury prevention

## Abstract

The present study examined and compared the isokinetic peak torque and the reciprocal ratios of the knee joint between young basketball and soccer players. An amount of 100 soccer and 100 basketball players took part in this study and were separated into five equal groups (n = 20), according to their chronological age (12, 13, 14, 15, and 16 years old). The absolute concentric (CON) and eccentric (ECC) peak torque of the knee flexor and extensor muscles (at 60°/s, 180°/s) were assessed using a Cybex Norm dynamometer, and the relative peak torque (per unit of body mass), as well as the conventional (CON/CON; ECC/ECC) and functional (CON/ECC; ECC/CON) ratios, were calculated. Data analysis indicated that the basketball players had higher absolute peak torque values than the soccer players throughout their developmental ages (*p* < 0.05). When the isokinetic peak torque values were normalized relative to body mass, no differences were observed between basketball and soccer players in any age group (*p* > 0.05). Additionally, no differences were observed in conventional and functional ratios between soccer and basketball players (*p* > 0.05). In conclusion, it appears that, during developmental ages (12–16 years old), the isokinetic strength profile (independent of body mass affecting absolute values) of knee extensor and flexor muscles develops similarly in basketball and soccer players.

## 1. Introduction

Basketball and soccer are two of the most widespread team sports all over the world during the developmental years. Different movements (e.g., sliding, jumps, kicks, duels, cutting maneuvers), in both sports, require high levels of lower-body strength [1,2,3,4], especially at the knee joint. There is also evidence that (a) the concentric and eccentric muscle strength of the knee joint is an important parameter to success in basketball and soccer [5,6], and (b) the muscular balance of the knee joint may decrease the predisposition of athletes to knee-related injuries [5,7]. Indeed, a low hamstring-to-quadriceps ratio (below the generally accepted values) may be associated with the presence of hamstring strain and/or anterior cruciate ligament (ACL) injury in young athletes [5,7,8,9]. Taking all the above into consideration, the systematic and reliable evaluation of knee extensors’ and flexors’ muscles, and, as a result, the calculation of conventional and functional reciprocal muscle group torque ratios, must be an integral part of the training and/or rehabilitation process during the developmental years. Isokinetic dynamometers have been widely used in sport and rehabilitation settings as safe (appropriate stabilization during the test), easily applicable following an appropriate familiarization, and acceptably reliable and valid devices for assessing lower limbs muscle strength and endurance in different joints of the human body during the developmental years [10,11,12]. Furthermore, isokinetic dynamometers have a great variety of indicative values and normative data in different populations and are often used as a reference standard (also called “Gold standard”) to compare other instruments of muscle strength measurement [10,13,14].

Earlier reports demonstrate age-related increases on muscle strength and power of lower limbs in trained boys [15,16,17]. More specifically, several studies showed significant age-related increases in concentric and eccentric peak torque and/or muscle group torque ratios (conventional-functional) of the knee [6,15,18,19], ankle [15,20], and hip [15,21,22] joints in young basketball or soccer players throughout the developmental years. An increase in muscle size, body dimensions, and neural maturation may account for these age-related effects on muscle strength and power throughout the developmental years [23]. There is a conception, nevertheless, that the pattern of improvement in muscle strength and power of lower and upper limbs may differ in populations with different characteristics, such as athletes vs. non-athletes, athletes of different sports, etc. [17,24,25,26]. Although basketball and soccer belong to team sports, they entail different skills, techniques, tactics, and muscle actions, influencing possibly the development of muscle strength in a different way.

Previous studies have examined the sport-related effect (basketball vs. soccer) on isokinetic peak torque of the knee joint in adult basketball and soccer players [27,28,29,30,31]; until now, however, there was limited information in young athletes [32]. Nonetheless, we believe that it is more important and reliable to examine sport-specific training effect on strength development during the developmental years, since young players rarely participate in weight training programs, in contrast to adult players. Therefore, all the differences that might be revealed in strength between athletes of different sports should be due to maturation and sport-specific training. In the scientific literature, only one study [32] has examined the sport-related effect on knee muscle strength of young basketball and soccer players. This study [32] has been performed in a single age-group (approximately 15 years old) and is limited to concentric muscle action, even though eccentric muscle strength of the knee joint is an important element for success in both team sports. Furthermore, to our knowledge, the effect of sport-specific training (basketball or soccer) on conventional and functional muscle group ratios of the knee joint have not been previously investigated during the developmental years. However, several studies in young and senior soccer or basketball players evaluated and created reference data for conventional and/or functional muscle group torque ratios of the knee joint [18,19,33,34,35,36,37]. The examination of possible sport-related effect on the development of reciprocal muscle group torque ratios is of utmost importance, as conventional and functional ratios of the knee flexor and extensor muscles may provide valuable information for knee joint stability and function, as well as for injury prevention and rehabilitation in young athletes [5,7,8,9].

Therefore, the main objective of this research was to investigate and compare the absolute and relative concentric and eccentric peak torque values of knee extensor and flexor muscles in young soccer and basketball players throughout the developmental years (12, 13, 14, 15, and 16 years old). We also investigated the sport-related effect (basketball vs. soccer) on conventional (CON/CON; ECC/ECC) and functional (CON/ECC; ECC/CON) reciprocal muscle group ratios at the knee joint.

## 2. Materials and Methods

### 2.1. Participants

One hundred soccer and one hundred basketball male players, aged from 12 to 16 years old, voluntarily took part in the present study. The participants, in each sport (basketball and soccer), were separated into five equal groups (n = 20) according to their chronological age: 12, 13, 14, 15, and 16 years old. All the participants: (a) involved in soccer or basketball training no less than three times/week for more than 12 months, (b) were healthy and did not have any lower limb injury for at least six months before the study, (c) did not perform any specific strength training program, and (d) did not have any preceding experience with isokinetic evaluation. Prior to the start of the study, children’s and adolescent’s health history and physical activity level were evaluated using a specific questionnaire [38]. Thereafter, the children’s parents were informed about the assessment process and filled in an informed consent form. The present research study was performed according to the ethical standards of the Declaration of Helsinki, and the Ethics Committee of the University of Thessaly granted the ethical approval. The somatometric and training characteristics of the participants (per age-group) are presented in Table 1.

### 2.2. Study Design

Prior to the start of the study, the participants were informed about the testing procedures and were acquainted with the isokinetic measurement. Furthermore, in the same day of familiarization, body height and body mass were assessed using a telescopic height rod (Seca model 220, Seca, Hamburg, Germany) and a calibrated physician’s scale (Seca model 755, Seca, Hamburg, Germany), respectively. After the acquaintance session, the participants came to the Training and Physical Conditioning Laboratory of the Department of Physical Education and Sports science of the University of Thessaly to perform isokinetic measurements. Before the isokinetic evaluation, the participants performed a standardized 15 min warm-up consisting of 7 min of stationary cycling (60 rpm at a workload of 60 W) at an exercise bike (Monark Ergomedic 874 E) and 8 min of static and dynamic stretching exercises for the lower limbs (especially for knee extensors and flexors muscles). All measurements were performed by the same investigator and were conducted at the same time of the day (9–12 a.m.) to avoid possible confounding effects of daily biorhythms.

### 2.3. Evaluation Procedures

A Cybex Norm dynamometer (Lumex Corporation, Ronkohoma, NY, USA) calibrated according to the manufacturer instructions was used for the isokinetic evaluation. Before the start of the measurement, the participants were positioned and stabilized on the isokinetic dynamometer, as previously described by Gerodimos et al. [18]. The axis of rotation of the dynamometer was carefully aligned with the approximate knee joint axis of rotation (posterior aspect of the lateral femoral condyle). The main testing protocol consisted of five maximum concentric and eccentric efforts of knee extensor and flexor muscles at angular velocities of 60°/s and 180°/s. Firstly, all the participants performed three to five primary familiarization trials of each type of muscle action and angular velocity where the range of motion of the measurement was defined from 0° full extension to 90° of knee flexion. The measurements were performed only in one leg. The tested leg was randomly determined in all cases to minimize the effect of possible leg asymmetries, as other studies have reported conflicting results regarding leg asymmetries. Some of them reported significant differences in force output between the two legs, while other studies reported no significant differences in force output or hamstring-to-quadriceps strength ratios between the two legs in young and adult individuals [19,33,39,40,41,42,43,44].

The eccentric and concentric tests were implemented separately in a randomized order, while a five-minute rest was given between different angular velocity and muscle action tests. The participants were instructed to move as hard as possible in both directions of movement. Visual feedback and stable verbal encouragement were provided during the isokinetic evaluation. The reliability of isokinetic measurements using the same testing protocols in young athletes is reported elsewhere [11]. The moments were corrected for the effects of gravity and the repetition with the highest moment was used for further analysis. The absolute peak torque (Nm) of knee extensor and flexor muscles was assessed, and the relative peak torque per unit of body mass (Nm/kg) was calculated (Nm/kg). The conventional (CON/CON and ECC/ECC) and functional (ECC/CON and CON/ECC) knee flexion (KF) to knee extension (KE) torque ratios were also calculated, as previously described by Gerodimos et al. [18].

### 2.4. Statistical Analysis

IBM SPSS 26.0 was used to analyze the data of the study. A statistical power analysis (software package GPower 3.0), prior to the start of the study, indicated that a total number of 100 basketball and 100 soccer players (20 participants in each age-group) would yield adequate power (>0.85) and a level of significance (<0.05). In addition, all the data were normally distributed according to the Kolomogorov-Smirnov test. Four-way ANOVAs (sport × age × muscle action × angular velocity, 2 × 5 × 2 × 2), with repeated measures on the factors “muscle action” and “angular velocity”, were used to analyze the data both for absolute and relative peak torque values of knee extensor and flexor muscles. Three-way ANOVAs (sport × age × angular velocity, 2 × 5 × 2), with repeated measures on the “angular velocity” factor, were used for data analysis of conventional and functional reciprocal ratios. In addition, two-way ANOVAs (sport × age; 2 × 5) were used to analyze somatometric and training characteristics. Sidak pair-wise comparisons were used to detect the significantly different means. All the data in the study are presented as means ± SD, and the significance level was set at *p* < 0.05.

## 3. Results

### 3.1. Somatometric—Training Characteristics

ANOVA results indicated significant “sport” (F_1,190_ = 51.38; *p* = 0.000 and F_1,190_ = 60.46; *p* = 0.000) and “age” (F_4,190_ = 57.28; *p* = 0.000 and F_4,190_ = 90.79; *p* = 0.000) main effects and a non-significant two-way interaction (F_4,190_ = 0.68; *p* = 0.61 and F_4,190_ = 0.14; *p* = 0.97) on body mass and body height, respectively. Basketball players demonstrated higher body height (*p* = 0.000) and body mass (*p* = 0.000) values compared with soccer players, irrespective of age. Pair-wise comparisons within age revealed that body height values increased with age (*p* = 0.000), with the exemptions of 14 vs. 15 years old and 15 vs. 16 years old, which did not differ in body height/mass values (*p* = 0.06–0.77), and of 12 vs. 13 years old, which did not differ in body mass values (*p* = 0.11). ANOVA results also demonstrated significant “age” main effect on training age (F_4,190_ = 14.02; *p* = 0.000) and training frequency (F_4,190_ = 11.44; *p* = 0.000), while non-significant “sport” main effect (F_1,190_ = 1.85–1.95; *p* = 0.22–0.25) and two-way interaction (F_4,190_ = 0.77–0.99; *p* = 0.41–0.54) were observed. Pair-wise comparisons within age revealed that training age and training frequency values increased with age (*p* = 0.000–0.003), while no year-by-year differences of 12 vs. 13, 13 vs. 14, 14 vs. 15 and 15 vs. 16 years old were observed in training age and training frequency values (*p* = 0.27–0.65).

### 3.2. Peak Torque

#### 3.2.1. Absolute Values

ANOVA statistical analyses showed significant “sport” (F_1,190_ = 14.19–20.23; *p* = 0.000), “age” (F_4,190_ = 78.19–102.68; *p* = 0.000), “muscle action” (F_1,190_ = 808.29–2026.16; *p* = 0.000) and “angular velocity” (F_1,190_ = 466.38–1903.39; *p* = 0.000) main effects and a non-significant four-way interaction (F_4,190_ = 0.41–1.14; *p* = 0.34–0.80) on absolute peak torque values. In more detail, basketball players demonstrated higher absolute peak torque values compared with soccer players in all age groups (*p* = 0.000). Furthermore, pair-wise comparisons within age demonstrated that absolute peak isokinetic torque values increased with age (*p* = 0.000), while no year-by-year differences were observed between 12 vs. 13, 14 vs. 15, and 15 vs. 16 years old (*p* = 0.35–0.55) in absolute torque values independent of angular velocity and muscle action. The only year by year difference were observed between 13 vs. 14 years old (*p =* 0.000). The peak torque values of knee extensor and flexor muscles were significantly (*p* = 0.000) lower at 180°/s compared to 60°/s independent of sport, age, and muscle action. Additionally, peak torque values of knee extensor and flexor muscles during concentric muscle action were significantly (*p* = 0.000) lower compared to those observed during eccentric muscle action, independent of sport, age, and angular velocity. The peak absolute torque values of knee extensor and flexor muscles at each sport, age, muscle action, and angular velocity are presented in Table 2.

#### 3.2.2. Relative Values

When the absolute peak torque values were adjusted for body mass, analysis of variance revealed a non-significant four-way interaction (F_4,190_ = 0.81–0.82; *p* = 0.52) and “sport” main effect (F_1,190_ = 1.89–2.25; *p* = 0.15–0.35), while significant “age” (F_4,190_ = 23.44–24.79; *p* = 0.000), “muscle action” (F_1,190_ = 938.96–1014.87; *p* = 0.000), and “angular velocity” (F_1,190_ = 517.51–961.29; *p* = 0.000) main effects were observed on relative peak torque values. Basketball and soccer players demonstrated similar relative torque values of knee extensor and flexor muscles (*p =* 0.55–0.85). Moreover, post-hoc analysis within the age main effect showed that, in 12- and 13-year-old basketball and soccer players, relative peak torque values of knee flexor and extensor muscles were significantly lower (*p* = 0.000) compared to those of 14, 15, and 16 years old counterparts. Meanwhile, no year-by-year differences were observed between 12 vs. 13, 14 vs. 15, 15 vs. 16 years old (*p* = 0.45–0.55). The only year-by-year differences were observed between 13 vs. 14 years old (*p =* 0.000). In addition, the relative torque values were significantly (*p =* 0.000) lower at fast angular velocity compared to slow angular velocity, independent of muscle action and age, and they were significantly lower during concentric muscle action compared to eccentric muscle action within all angular velocities and age-groups (*p =* 0.000). The relative peak torque values of knee extensor and flexor muscles at each sport, age, muscle action, and angular velocity are presented in Table 3.

### 3.3. Conventional and Functional Reciprocal Ratios

ANOVAs revealed non-significant interaction (F_4,190_ = 0.35–1.23; *p* = 0.30–0.85) or main effects of “sport” (F_1,190_ = 0.01–1.02; *p* = 0.32–0.99) and “age” (F_4,190_ = 1.78–2.44; *p* = 0.10–0.20) on conventional and functional reciprocal muscle group torque ratios of the knee joint, both at slow and fast angular velocities. Nevertheless, a significant “angular velocity” main effect on both conventional (F_1,190_ = 16.95–68.34; *p* = 0.000) and functional (F_1,90_ = 172.07–765.27; *p* = 0.000) muscle group torque ratios was reported. Specifically, concentric and eccentric conventional (CON/CON and ECC/ECC) reciprocal ratio values were significantly (*p =* 0.000) higher at fast angular velocity compared to those reported at slow angular velocity within all age groups and sports. Likewise, ECC/CON functional ratio values were significantly higher at fast angular velocity compared to those reported at slow angular velocity; whereas, CON/ECC functional ratio values were significantly lower at 180^o^/s compared to those observed at 60°/s, irrespective of age and sport (*p =* 0.000). The conventional (CON/CON; ECC/ECC) and functional (ECC/CON; CON/ECC) reciprocal muscle group ratio values per sport, age, and angular velocity are presented in Table 4.

## 4. Discussion

This study investigated the sport-related (basketball vs. soccer) differences in the isokinetic peak torque and the reciprocal ratios of the knee joint throughout the developmental years (12–16 years old). The main results of the present study indicated that: a) basketball players revealed higher absolute peak torque values in knee extensor and flexor muscles than soccer players and b) basketball and soccer players demonstrated similar relative peak torque values and similar values in both conventional and functional ratios throughout the developmental years. It seems that the differences observed in somatometric characteristics (body mass and body height) between basketball and soccer players affected the absolute peak torque values of knee extensors and flexors muscles. To our knowledge, no previous study compared the isokinetic peak torque of knee extensor and flexor muscles in youth basketball and soccer players throughout the developmental years (12–16) over a range of muscle actions (concentric and eccentric).

In the scientific literature, there is only one study [32], which compared the concentric peak torque at knee extensor and flexor muscles (60–240°/s) between young basketball and soccer players, in a single age-group of 15–16 years old adolescents, reporting similar results to that of the present study. Indeed, Erdemir et al. [32] found that the absolute peak torque values in knee extensor and flexors muscles were significantly greater in adult basketball than in soccer players. Likewise, in accordance with our findings, no significant differences were noted when peak torque values were expressed per body mass. The greater absolute peak torque values may be attributed to the greater body mass values of the basketball players compared to soccer players. The results of this study and those of Erdemir et al. [32] support the findings of previous studies, which reported that body mass has a significant effect on peak torque values of knee extensor and flexor muscles [45]. In the same context, Housh et al. [46] compared the age-related increases in muscle strength with changes in body mass and fat free mass in young wrestlers and commented that the strength increases cannot be attributed only to changes in body mass. In more detail, there is evidence that the increase in absolute strength during the developmental years has been attributed to: (a) changes in muscle mass per unit of fat free weight [46], (b) changes to neuromuscular responses, and (c) leg musculature, which take place as the child grows up [46,47].

The only ambivalent result between our study and that of Erdemir et al. [32] has been reported in peak torque ratio values, where basketball players reported greater values in the left limb at 60°/s than soccer players. The authors [32] reported that the difference in peak torque ratio values in the left limb was due to bilateral strength differences between legs in soccer players, which were not found in basketball players. Additionally, previous studies, in adult males, which examined differences in peak torque values between soccer and basketball players, reported contradictory results [27,28,29,30,31]. More specifically, some of them found significant differences between basketball and soccer players, and some of them found the same levels of peak torque values.

In the present study, we also documented an age-related increase, with some exceptions, both in concentric and eccentric absolute and relative peak torque values in young basketball and soccer players. These results follow the findings of previous studies that also observed an age-related increase in concentric and/or eccentric peak torque values of the knee [6,15,18,19], hip [15,21,22], and ankle [15,20] joints during growth and development both in young basketball and soccer players. The most pronounced increase in isokinetic peak torque values was observed from 12–13 years old to 14–16 years old, which mirrors the maturation sparks observed within these age intervals. The age-related increases in isokinetic peak torque values may be attributed to different factors, such as the endocrine alterations (differences in hormone production), neural maturation, and myelination of nerve fibers that happen during growth and development, in addition to the fact that pubertal boys can voluntarily activate a higher percentage of available motor units compared to pre-pubertal boys [48,49]. In the present study, no year-by-year differences (12 vs. 13, 14 vs. 15, 15 vs. 16 years old) were observed in most cases in peak torque values, as previously described and in other studies in different joints [18,20]. The non-significant year by year differences may be attributed to the similar maturation stage of these year-by-year groups (although this was not evaluated in the present study), as well as in the training characteristics (training age and training frequency), where no significant year by year differences were observed. The only year-by-year difference, both in absolute and relative peak torque values, was observed between 13 vs. 14 years old, where there was a pronounced increase in strength, maybe due to the onset of puberty and maturation spark. We also found that absolute and relative peak torque values of knee extensor and flexor muscles are significantly lower during concentric compared to eccentric muscle action and significantly lower at high angular velocities compared to low angular velocities. Our above findings strengthen the results of previous studies in the scientific literature [18,20,22].

Additionally, in our study, we demonstrated that conventional and functional muscle group torque ratios did not significantly change throughout the developmental years, both in basketball and soccer players. The results of the present study are in accordance with the findings of previous studies in youth athletes, demonstrating no significant age-related effects on conventional and functional muscle group torque ratios values of the knee, ankle, and shoulder joints in young athletes [18,20,22,50]. Finally, the conventional ratio (CON/CON and ECC/ECC) and the ECC/CON functional ratio values were lower in slow angular velocity (60°/s) compared to high angular velocity (180°/s); whereas, the CON/ECC functional ratio values were higher at low angular velocity (60°/s) compared to high angular velocity (180°/s). The aforementioned findings are in agreement with the results of previous studies that examined the effect of angular velocity on conventional and functional ratios at the knee or hip joints [18,22,51]. There is evidence that, as angular velocity of the movement increases, the maximum torque generation capacity of the antagonist musculature increases, especially when the antagonists lengthen and the agonist muscles produce concentric force during the movement [18,22,51]. This may negatively affect knee joint stabilization, especially if one considers the high magnitude forces exerted due to the lengthening action of the knee flexor muscles.

The present study has some limitations that could affect its outcomes, and, as a result, their generalization. First of all, the findings of this study are clearly limited to young basketball and soccer players (12–16 years old). Future studies could examine the sport-related effect on isokinetic peak torque values in other age-groups or in other team or individual sports. Furthermore, future studies should examine the effect of maturation stage, the effect of sex, as well as the effect of specific strength training program, in conjunction with the sport-related effect on isokinetic peak torque values and reciprocal ratios of the knee joint. Another limitation of this study is the fact that the measurements were performed only in one leg. The effect of leg preference on isokinetic peak torque has been a matter of debate because some studies reported that the preferred leg was stronger than the non-preferred leg, and others reported no differences between legs. Future studies should examine the effect of leg preference in conjunction with sport-related effect on isokinetic peak toque values, as well as on reciprocal ratios. Finally, the results of this study are limited to the testing protocol used (i.e., angular velocities) for the isokinetic evaluation of knee extensors and flexors muscles.

## 5. Conclusions

In conclusion, from the results of the present study, it seems that the basketball players have higher absolute peak torque values of the knee extensor and flexor muscles than the soccer players throughout the developmental ages (from 12 to 16 years old). It should be mentioned that, when the isokinetic peak torque values were normalized relative to body mass, no differences were detected between basketball and soccer players in any age-group. It seems that the differences in somatometric characteristics (especially in body mass) between basketball and soccer players affect the absolute peak torque values of knee extensors and flexors muscles. Moreover, no differences were observed in both conventional and functional ratios between soccer and basketball players. Thus, it appears that, during developmental ages (12–16 years old), the isokinetic peak torque (independent of body mass affecting absolute values) of knee extensor and flexor muscles develops similarly both in basketball and soccer players. The data presented in this study serve to provide a comprehensive profile of isokinetic peak torque of knee extensors and flexors muscles to assist both coaches and health professionals for talent selection, as well as for development of appropriate training programs for performance enhancement and rehabilitation. Furthermore, the examination of possible sport-related effect on the development of reciprocal muscle group torque ratios is of crucial clinical importance, as muscle group torque ratios (conventional and functional) of the knee flexor and extensor muscles may provide significant information for knee joint stability and function, as well as for injury prevention and rehabilitation in young athletes.

## Figures and Tables

**Table 1:** Somatometric and training characteristics of the participants per sport and age-group (mean ± sd).

Age Groups						
	Sport	12 Years	13 Years	14 Years	15 Years	16 Years
Body mass (kg)	Soccer	41.4 ± 7.6	46.0 ± 9.8	57.4 ± 7.4	62.5 ± 8.8	68.9 ± 5.7
	Basket	49.5 ± 11.7	55.8 ± 10.5	68.7 ± 10.5	75.5 ± 12.9	75.5 ± 9.4
Body height (cm)	Soccer	149 ± 8.3	155 ± 8.4	168 ± 7	171 ± 5.6	176 ± 5.9
	Basket	156 ± 7.4	163 ± 8.8	176 ± 4.6	180 ± 10.9	185 ± 8.2
Training age (years)	Soccer	3.2 ± 1.4	4.0 ± 1.9	4.5 ± 1.4	5.7 ± 1.9	5.8 ± 1.7
	Basket	3.0 ± 1.3	3.8 ± 1.5	4.2 ± 1.6	5.6 ± 1.8	5.7 ± 1.8
Training frequency (times/week)	Soccer	3.6 ± 0.9	4.3 ± 0.9	4.7 ± 0.9	5.5 ± 1.8	5.9 ± 1.6
	Basket	3.5 ± 0.8	4.2 ± 0.8	4.5 ± 0.9	5.3 ± 1.5	5.9 ± 1.8

**Table 2:** Maximum isokinetic concentric and eccentric absolute peak torque (Nm) of the knee flexors and extensors muscles at 60°/s and 180°/s angular velocities, in different sports and age groups.

Absolute Peak Torque Values of Knee Flexors (Nm)								
Velocity	Muscle Action	Sport	Age	12	13	14	15	16
60°/s	Con	Soccer	Mean	54.8	69.0	96.9	110.8	120.1
			SD	12.8	18.3	16.1	18.3	18.4
		Basket	Mean	63.0	79.0	120.4	124.7	132.1
			SD	14.1	15.8	24.0	22.5	21.3
	Ecc	Soccer	Mean	74.9	89.2	121.1	142.6	153.5
			SD	19.3	19.5	25.7	28.7	28.2
		Basket	Mean	83.4	98.9	152.0	161.4	168.8
			SD	18.0	23.7	32.8	34.9	26.9
180°/s	Con	Soccer	Mean	44.0	51.7	75.2	83.4	96.1
			SD	10.2	16.4	14.9	12.2	17.2
		Basket	Mean	47.0	59.9	91.8	90.3	99.6
			SD	12.0	12.8	18.3	17.6	18.2
	Ecc	Soccer	Mean	75.2	86.3	127.3	142.0	151.5
			SD	20.2	22.6	28.0	32.9	25.3
		Basket	Mean	81.0	90.6	145.1	151.3	158.1
			SD	16.2	22.1	31.7	30.8	25.0
**Absolute Peak Torque Values of Knee Extensors (Nm)**								
**Velocity**	**Muscle Action**	**Sport**	**Age**	**12**	**13**	**14**	**15**	**16**
60°/s	Con	Soccer	Mean	88.3	108.8	146.5	160.5	192.0
			SD	20.8	27.6	24.5	27.4	31.8
		Basket	Mean	99.7	123.5	170.4	188.4	203.1
			SD	23.1	25.8	29.2	27.2	21.7
	Ecc	Soccer	Mean	114.8	139.8	182.4	213.4	247.8
			SD	29.1	32.0	37.3	41.1	42.6
		Basket	Mean	133.8	155.2	217.3	250.6	257.0
			SD	28.0	32.6	53.9	50.2	43.7
180°/s	Con	Soccer	Mean	66.3	78.4	108.3	119.8	143.3
			SD	15.3	19.6	17.6	16.9	23.7
		Basket	Mean	71.0	87.5	127.4	135.0	151.6
			SD	16.7	18.6	30.5	24.4	21.5
	Ecc	Soccer	Mean	106.8	128.0	174.9	186.7	227.4
			SD	23.9	34.4	35.2	41.5	36.6
		Basket	Mean	116.4	132.0	195.1	218.4	220.1
			SD	29.9	22.5	35.9	32.6	36.5

**Table 3:** Maximum isokinetic concentric and eccentric relative peak torque (Nm/Kg) of the knee flexors and extensors muscles at 60°/s and 180°/s angular velocities in different sports and age groups.

**Relative Peak Torque Values of Knee Flexors (Nm/kg)**								
**Velocity**	**Muscle Action**	**Sport**	**Age**	**12**	**13**	**14**	**15**	**16**
60°/s	Con	Soccer	Mean	1.32	1.50	1.70	1.78	1.75
			SD	0.15	0.24	0.22	0.23	0.24
		Basket	Mean	1.29	1.44	1.73	1.66	1.74
			SD	0.21	0.29	0.33	0.22	0.18
	Ecc	Soccer	Mean	1.81	1.95	2.11	2.27	2.23
			SD	0.36	0.20	0.37	0.34	.35
		Basket	Mean	1.73	1.79	2.18	2.14	2.24
			SD	0.34	0.40	0.44	0.34	0.32
180°/s	Con	Soccer	Mean	1.06	1.13	1.31	1.34	1.39
			SD	0.13	0.28	0.20	0.16	0.20
		Basket	Mean	0.96	1.08	1.32	1.20	1.31
			SD	0.16	.20	0.26	0.17	0.16
	Ecc	Soccer	Mean	1.81	1.87	2.22	2.26	2.20
			SD	0.29	0.25	0.30	0.32	0.31
		Basket	Mean	1.69	1.64	2.10	2.01	2.10
			SD	0.37	0.37	0.34	0.28	0.30
**Relative Peak Torque Values of Knee Extensors (Nm/kg)**								
**Velocity**	**Muscle Action**	**Sport**	**Age**	**12**	**13**	**14**	**15**	**16**
60°/s	Con	Soccer	Mean	2.14	2.36	2.55	2.57	2.78
			SD	0.29	0.29	0.27	0.24	0.35
		Basket	Mean	2.05	2.24	2.47	2.52	2.75
			SD	0.36	0.35	0.34	0.32	0.34
	Ecc	Soccer	Mean	2.77	3.06	3.19	3.39	3.59
			SD	0.41	0.37	0.47	0.49	0.49
		Basket	Mean	2.77	2.84	3.17	3.36	3.41
			SD	0.51	0.64	0.61	0.67	0.56
180°/s	Con	Soccer	Mean	1.60	1.71	1.89	1.92	2.07
			SD	0.17	0.24	0.18	0.13	0.25
		Basket	Mean	1.45	1.58	1.84	1.80	2.01
			SD	0.20	0.29	0.24	0.21	0.22
	Ecc	Soccer	Mean	2.58	2.77	3.06	2.99	3.29
			SD	0.30	0.33	0.49	0.49	0.39
		Basket	Mean	2.41	2.41	2.83	2.93	2.92
			SD	0.55	0.48	0.44	0.57	0.51

**Table 4:** Knee flexor (KF)/extensor (KE) ratios (%) at 60°/s and 180°/s angular velocities in different sports and age groups.

Ratios	Sport	Velocity	Age	12	13	14	15	16
CON_KF_/CON_KE_	Soccer	60°/s	Mean	62.6	63.5	66.4	69.6	63.7
			SD	8.6	7.0	5.4	8.7	7.1
		180°/s	Mean	66.7	65.8	69.4	70.2	67.5
			SD	8.5	9.0	7.6	8.9	8.4
	Basketball	60°/s	Mean	63.8	64.4	70.4	66.5	65.1
			SD	9.1	5.9	9.9	9.2	8.2
		180°/s	Mean	66.6	68.7	71.6	67.4	65.6
			SD	10.7	6.9	10.5	10.4	6.6
ECC_KF_/ECC_KE_	Soccer	60°/s	Mean	66.1	64.3	67.0	67.3	63.0
			SD	10.9	7.6	10.5	11.3	9.9
		180°/s	Mean	70.6	67.9	73.5	77.4	66.9
			SD	12.5	8.4	11.6	15.6	6.7
	Basketball	60°/s	Mean	62.8	64.1	69.3	64.7	66.2
			SD	8.5	11.8	11.7	9.0	6.6
		180°/s	Mean	71.3	68.3	74.6	69.8	72.7
			SD	11.6	10.6	11.8	9.2	9.5
ECC_KF_/CON_KE_	Soccer	60°/s	Mean	86.1	83.1	82.7	88.7	81.2
			SD	14.8	9.9	11.6	16.5	15.9
		180°/s	Mean	114.1	111.0	118.3	117.9	107.1
			SD	18.9	16.2	17.7	17.6	17.6
	Basketball	60°/s	Mean	85.1	80.4	88.3	85.2	83.5
			SD	15.3	11.8	17.5	11.0	12.3
		180°/s	Mean	116.3	105.0	114.4	112.4	105.2
			SD	17.1	18.6	17.5	15.1	16.0
CON_KF_/ECC_KE_	Soccer	60°/s	Mean	48.8	49.4	54.1	53.5	49.0
			SD	9.7	7.5	8.3	9.2	6.8
		180°/s	Mean	41.4	40.8	44.0	46.3	42.6
			SD	5.5	9.4	9.9	9.8	5.5
	Basketball	60°/s	Mean	47.3	51.7	56.2	51.0	52.3
			SD	6.1	9.3	9.2	10.2	10.2
		180°/s	Mean	41.5	45.6	47.4	42.2	46.4
			SD	11.1	7.7	11.1	8.3	10.9

## Data Availability

Data are unavailable due to privacy or ethical restrictions.
